# Supplementary material for: The causal relationship between the human gut microbiota and pyogenic arthritis: a Mendelian randomization study
Source: Front Cell Infect Microbiol. 2024 Nov 26;14:1452480. doi: 10.3389/fcimb.2024.1452480 (PMC11629706; doi:10.3389/fcimb.2024.1452480)
Supplement: Supplementary file 1 [file DataSheet1.zip › mendelian test/ebi-a-GCST90027772.csv_forest.pdf]

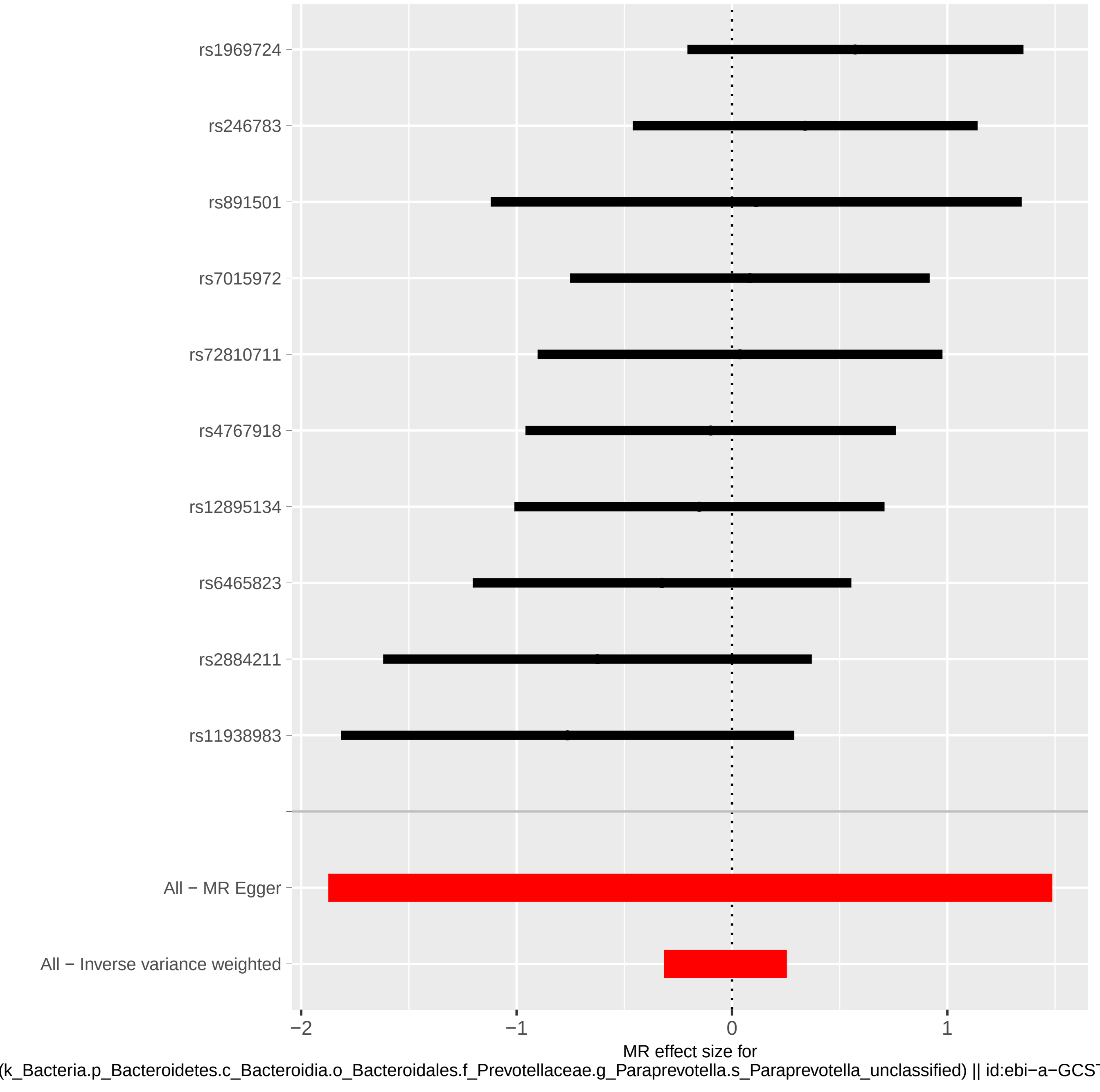

rs1969724
rs246783
rs891501
rs7015972
rs72810711
rs4767918
rs12895134
rs6465823
rs2884211
rs11938983
All − MR Egger
All − Inverse variance weighted
−2
−1
0
1
MR effect size for
(k_Bacteria.p_Bacteroidetes.c_Bacteroidia.o_Bacteroidales.f_Prevotellaceae.g_Paraprevotella.s_Paraprevotella_unclassified) || id:ebi−a−GCST
